# Could 4-Week Walnut Consumption Influence Oxidative and Inflammatory Status in Middle-Aged Adults with Cardiometabolic Risk Factors? Findings from a Randomized Controlled Trial

**DOI:** 10.3390/nu17172826

**Published:** 2025-08-30

**Authors:** Letiția Mateș, Ionel Fizeșan, Daniel-Corneliu Leucuța, Andreea-Elena Petru, Dana Maria Muntean, Doina Albert-Ani, Oana Andreea Alexa, Marius Emil Rusu, Lorena Filip, Daniela-Saveta Popa

**Affiliations:** 1 Department of Toxicology, Faculty of Pharmacy, Iuliu Hatieganu University of Medicine and Pharmacy, 8 Victor Babes, 400012 Cluj-Napoca, Romania; micu.letitia@umfcluj.ro (L.M.); ionel.fizesan@umfcluj.ro (I.F.); petruandreeaelena@gmail.com (A.-E.P.); alexaoana9@gmail.com (O.A.A.); dpopa@umfcluj.ro (D.-S.P.); 2 Department of Medical Informatics and Biostatistics, Faculty of Medicine, Iuliu Hatieganu University of Medicine and Pharmacy, 6 Pasteur Street, 400012 Cluj-Napoca, Romania; 3 Department of Pharmaceutical Technology and Biopharmaceutics, Faculty of Pharmacy, Iuliu Hatieganu University of Medicine and Pharmacy, 8 Victor Babes, 400012 Cluj-Napoca, Romania; dana.muntean@umfcluj.ro (D.M.M.); rusu.marius@umfcluj.ro (M.E.R.); 4 Clinic of Occupational Medicine, Cluj County Emergency Clinical Hospital, 3-5 Clinicilor Street, 400006 Cluj-Napoca, Romania; doinapirvalbert@gmail.com; 5 Department of Bromatology, Hygiene, Nutrition, Faculty of Pharmacy, Iuliu Hatieganu University of Medicine and Pharmacy, 6 Pasteur Street, 400349 Cluj-Napoca, Romania; lfilip@umfcluj.ro; 6 Academy of Romanian Scientists (AOSR), 3 Ilfov Street, 050044 Bucharest, Romania

**Keywords:** *Juglans regia*, oxidative stress, inflammation, catalase, glutathione peroxidase, total antioxidant capacity, interleukins, tumor necrosis factor alpha, inflammaging

## Abstract

Background: Oxidative stress (OS) and inflammation are interconnected processes with significant roles in various chronic diseases, particularly those associated with aging, such as metabolic syndrome (MetS). Recent evidence suggests that walnuts (from *Juglans regia* L.), due to their rich content of phytochemicals, have antiaging potential by attenuating OS and chronic low-grade inflammation, known as inflammaging. Objectives: We aimed to assess the impact of daily walnut consumption for 4 weeks on biomarkers of OS and inflammation in a cohort of middle-aged individuals at risk of developing MetS. Methods: In this crossover randomized controlled trial (RCT), 22 participants (mean age: 48.81 ± 4.3 years) underwent two 28-day dietary interventions separated by a one-month washout period. One intervention period included daily consumption of 45 g of walnuts, while the other (control period) involved a normal-calorie diet without walnuts. Catalase (CAT) and glutathione peroxidase (GPx) activities, total antioxidant capacity (TAC), and interleukin (IL-1β, IL-6, IL-8) and tumor necrosis factor alpha (TNF-α) levels were determined from serum before and after each intervention period. Results: Assessment of changes obtained for the selected biomarkers following the walnut and control-diet periods (final-baseline) showed slight changes, but without any statistical significance, among the 20 participants included in the analysis. Conclusions: This first RCT targeting a group of middle-aged adults at risk of developing MetS shows that short-term (4 weeks) daily walnut consumption did not significantly alter oxidative stress and inflammation parameters, thus potentially contributing to the maintenance of cellular homeostasis. Further research is needed to investigate the impact of daily walnut consumption over a longer period (>3 months) on oxidative and inflammatory status in the middle-aged population and its potential to positively impact MetS biomarkers.

## 1. Introduction

Aging is the primary risk factor for age-related diseases (ARDs), including obesity, type 2 diabetes (T2D), cardiovascular disease (CVD), cancer, and neurodegenerative disorders [1]. In recent decades, the global trend toward population aging has coincided with a widespread increase in obesity and related metabolic disorders, particularly T2D [2,3]. Adipose tissue dysfunction, a hallmark of aging, drives systemic metabolic alterations, including insulin resistance, ectopic lipid accumulation, and chronic inflammation, elevating the risk of obesity and T2D in older adults [1].

Metabolic syndrome (MetS), characterized by a cluster of cardiovascular risk factors such as obesity, dyslipidemia, hypertension, and impaired glucose tolerance, is a global public health concern [4]. Oxidative stress and chronic low-grade inflammation are central mechanisms underlying MetS progression. When adipocytes exceed their storage capacity due to excessive energy supply, hypertrophy occurs, leading to the release of pro-inflammatory cytokines such as interleukin-1 (IL-1), interleukin-6 (IL-6), and tumor necrosis factor alpha (TNF-α) [5,6]. This triggers systemic inflammation, with TNF-α inducing insulin resistance by inhibiting insulin receptor phosphorylation, thereby disrupting insulin signaling and contributing to dyslipidemia and T2D [7,8]. Oxidative stress, driven by adipose tissue expansion, hypoxia, and the release of reactive oxygen and nitrogen species (ROS and RNS, respectively) during phagocytosis, exacerbates inflammation and insulin resistance [4]. This redox imbalance generates advanced glycation end-products (AGEs) and advanced lipoxidation end-products (ALEs), further promoting MetS components. In hypertension, oxidative stress correlates with endothelial dysfunction and elevated blood pressure, impairing vasodilation and promoting vascular damage [5,9]. Emerging evidence highlights the role of diet, particularly healthy plant-based foods rich in bioactive compounds [10,11,12].

Walnuts (from *Juglans regia* L.), known for their high content of polyunsaturated fatty acids (PUFAs), polyphenols, and other bioactive compounds, have demonstrated antioxidant, anti-inflammatory, and antiaging properties [13,14,15]. Key phenolic constituents in walnuts, including ellagic acid, tannins, and ellagitannins, have been shown to mitigate oxidative stress and inflammation by scavenging free radicals and modulating nuclear pathways [16,17]. Additionally, walnuts are rich in essential nutrients, including vitamins, minerals, and amino acids, which contribute to their health-promoting effects [18,19]. Clinical trials have identified ellagitannins as one of the primary bioactive compounds in walnuts, which, upon ingestion, are metabolized into ellagic acid and urolithins, exerting systemic antioxidant and anti-inflammatory effects [20,21,22]. These compounds could prevent oxidative damage through multiple mechanisms of action, including activation of the Nrf2/ARE signaling pathway; enhancing the gene expression and activity of antioxidant enzymes, such as catalase (CAT), superoxide dismutase (SOD), and glutathione peroxidase (GPx); and neutralizing ROS [23,24,25]. Furthermore, walnut kernels contain approximately 18–24% protein and are a rich source of essential amino acids, including glutamic acid, arginine, and aspartic acid, which play crucial roles in detoxification, neurotransmitter synthesis, and metabolic regulation [26]. Enzymatic hydrolysis of walnut protein yields bioactive peptides that exhibit a broad range of biological activities, notably antioxidant, anti-inflammatory, neuroprotective, and antihypertensive effects [27]. These low-molecular-weight peptides, characterized by a high content of hydrophobic and aromatic amino acids such as leucine, phenylalanine, histidine, tryptophan, and tyrosine, demonstrate the capacity to scavenge free radicals, inhibit key inflammatory signaling pathways, including NF-κB and TLR4-MyD88, and modulate lipid metabolism. Furthermore, specific peptides derived from walnut proteins have been shown to enhance cognitive functions, attenuate oxidative stress, and exert neuroprotective effects in models of neurodegenerative diseases such as Alzheimer’s disease [28].

Despite the growing body of research on walnuts, significant gaps remain in understanding their short-term effects on oxidative and, in particular, inflammatory biomarkers, especially in middle-aged adults at risk of MetS, according to the results of a previously conducted and recently published meta-analysis [29]. Previous studies have primarily focused on long-term interventions or specific populations, leaving the immediate cellular responses to walnut consumption underexplored. Addressing these gaps is crucial for developing targeted dietary recommendations to manage oxidative stress and inflammation in individuals at risk for MetS.

To our knowledge, this is the first randomized, controlled, crossover trial (RCT) aiming to investigate whether short-term (4 weeks) daily consumption of walnuts, within a normocaloric diet, could improve oxidative and inflammatory cellular status in middle-aged adults at risk of MetS. Specifically, this study evaluated changes in key biomarkers of oxidative stress (e.g., CAT, GPx, total antioxidant capacity (TAC)) and inflammation (e.g., IL-6, IL-8, IL-1β, TNF-α) following walnut intervention, providing insights into the potential role of walnuts as a dietary strategy for managing MetS-related oxidative stress and inflammation.

## 2. Materials and Methods

This study is part of a larger research project (clinical trial registration number ISRCTN17119161, registration date: 26 September 2024), with several results previously published [30]. The research was conducted in accordance with the CONSORT 2010 statement criteria, with extensions for randomized crossover trials [31].

### 2.1. Study Design

The present RCT was designed to evaluate the effects of daily walnut consumption on several oxidative stress and inflammatory parameters. To prevent any residual effects, the trial was structured into two 28-day intervention periods, each followed by a one-month washout period (31 days). For the first intervention period, participants were randomly assigned to either a daily serving of walnut kernels (45 g per day) or a regular normocaloric diet without walnuts as a control. The 45 g of walnut per person per day corresponded to a range of approximately 0.45 to 0.63 g/kg/day, with a mean of 0.52 g/kg/day. The second period involved participants receiving the opposite treatment.

### 2.2. Participants

Study participants were recruited from Cluj-Napoca, Romania, and screened for eligibility according to previously established criteria [30]. Briefly, key inclusion criteria included men and women aged 40–65 years with at least one MetS-specific parameter (abdominal obesity, dyslipidemia, dysglycemia, or hypertension). Exclusion criteria included allergies to nuts, restrictive diets, eating disorders, chronic conditions, pregnancy, smoking, chronic alcohol consumption, and recent use of medications or dietary supplements.

### 2.3. Interventions

The dietary intervention followed a specific protocol [30]. In brief, walnuts were sourced from Satu Mare County, Romania, and participants were assigned to either a walnut intervention group (45 g/day, *n* = 11) or a control group (nut-free, *n* = 9) for 28 days. All participants received individualized dietary recommendations based on current dietary guidelines [32] to maintain their weight. The method for assessing dietary intake and calculating caloric requirements was also described in detail. Participants were instructed to avoid nuts, seeds, nut/seed butters, and related products, as well as supplements with anti-inflammatory and antioxidant agents (e.g., fish oil, resveratrol, curcumin, vitamin C, selenium, zinc), for at least two weeks before the initial assessment and throughout the study. Consistent with the crossover design outlined, a 31-day washout period separated the intervention phases to allow for the clearance of any residual effects from the initial treatment.

### 2.4. Randomization and Blinding

Participants were randomized to either the intervention or control group using a computer-generated schedule with varying block sizes. Allocation concealment was ensured by a researcher distributing walnut packets according to patient ID, while participant blinding was not feasible due to the nature of the intervention. Laboratory technicians and data analysts were blinded to group assignments.

### 2.5. Data Collection and Outcome Measures

Four evaluations were conducted, with measurements at the beginning and the end of each four-week intervention period. The outcomes for this study consisted of measurements of (1) oxidative stress parameters—TAC (mmol/L), CAT (U/mL), and GPx (U/L)—and (2) inflammation biomarkers—IL-1β (pg/mL), IL-6 (pg/mL), IL-8 (pg/mL), and TNF-α (pg/mL).

Standardized protocols were strictly followed throughout the study to ensure consistent and accurate measurements. For each study participant, two venous blood samples (10 mL) were collected by a medical team (from the laboratory of the Occupational Medicine Clinic, Cluj County Emergency Clinical Hospital, Cluj-Napoca, Romania) after a 12 h fasting period. For the evaluation of TAC, CAT, and GPx, the blood samples were processed in tubes containing a coagulation activator. Blood samples for IL-1β, IL-6, IL-8, and TNF-α analysis were collected in tubes that additionally included a gel separator to isolate serum for cytokine quantification. All samples were immediately transferred to the laboratory of the Department of Toxicology, Faculty of Pharmacy, Iuliu Hațieganu University of Medicine and Pharmacy, Cluj-Napoca, Romania, and the blood was allowed to clot at room temperature (18–24 °C) for 30 min. Serum fractions were separated by centrifugation (Hettich, Micro 22R Andreas Hettich GmbH & Co., Tuttlingen, Germany) at 3000 rpm and 4 °C for 15 min, in accordance with a previously published protocol [33]. The serum aliquots (500 µL) were stored in vials at −80 °C prior to analysis.

#### 2.5.1. Determination of Oxidative Stress Parameters

Given the established role of oxidative stress in the pathophysiology of MetS, TAC, CAT, and GPx were selected as a complementary panel of relevant biomarkers to comprehensively assess the potential of walnut consumption to modulate redox balance and improve antioxidant defenses in this at-risk population [34]. TAC provides an integrated measure of overall antioxidant capacity, while CAT and GPx quantify specific enzymatic defenses against ROS, particularly H_2_O_2_ [35,36].

Reagents: All standards and reagents were of analytical grade. 2,2-Azinobis(3-ethylbenzothiazoline-6-sulfonate) (ABTS), acetic acid, ammonium acetate, bovine serum albumin, calcium chloride, copper (II) chloride dihydrate, dipotassium phosphate (K_2_HPO_4_), ethylenediaminetetraacetic acid disodium salt dihydrate (EDTA), hydrochloric acid, sodium dihydrogen phosphate dihydrate (NaH_2_PO_4_·2H_2_O), glutathione, neocuproine (2,9-dimethyl-1,10-phenanthroline), hydrogen peroxide (30%), disodium hydrogen phosphate dihydrate (Na_2_HPO_4_·2H_2_O), sodium azide, sodium hydroxide, sodium carbonate, sodium nitrate, sodium hexametaphosphate, tert-butyl hydroperoxide, trichloroactetic acid, and 6-hydroxy-2,5,7,8-tetramethylchroman-2-carboxylic acid (Trolox) were purchased from Sigma-Aldrich (Schnelldorf, Germany). The water utilized in our investigation was ultrapure, obtained from a Milli-Q ultrapure water system (Millipore, Burlington, MA, USA).

##### Total Antioxidant Capacity by Trolox Equivalent Antioxidant Capacity (TEAC) Assay

Total antioxidant capacity (TAC) was evaluated using an adapted spectrophotometric method [37]. This assay is based on the ability of antioxidants in the sample to reduce the blue-green radical cation 2,2′-azino-bis(3-ethylbenzothiazoline-6-sulfonic acid) (ABTS•+), resulting in an absorbance decrease measured at 660 nm. The rate of ABTS•+ reduction is directly proportional to both the concentration and antioxidant potential of the present antioxidants. In brief, 200 µL of ABTS radical solution was mixed with 20 µL of sample, followed by 6 min of incubation, the addition of 20 µL of methanol for deproteinization, and 10 min of centrifugation at 5000 rpm (Hettich, Micro 22R, Andreas Hettlich GmbH & Co. KG, Tuttlingen, Germany). The absorbance was measured at 760 nm using a Jasco V-530 UV/Vis spectrophotometer (, Tokyo, Japan). The scavenging activity of Trolox (as external standard) against the ABTS radical cation was measured and used to create the Trolox calibration curve. TAC values are reported as mmol Trolox equivalents/L.

##### Catalase Activity Assessment

Catalase activity was assessed spectrophotometrically by quantifying the rate of hydrogen peroxide (H_2_O_2_) decomposition at 240 nm, following a standard method [38]. The assay, performed at 25 °C, involved monitoring the decrease in absorbance over 1 min in a 2.0 mL reaction mixture containing 50 mM potassium phosphate buffer (pH 7.0), sample, and H_2_O_2_ (10 µmol/mL final concentration). One unit of catalase activity was defined as the amount of enzyme required to degrade 1 µmol of H_2_O_2_ per minute under these conditions.

Catalase activity was calculated using the following formula:

[U/mL] = (ΔA × Vt)/(εmM × d × Vp)

where ΔA = A1 − A2 (change in absorbance at 240 nm); Vt = 2.0 mL (total reaction volume); εmM = 0.0436 mM × 1 cm^−1^ (molar extinction coefficient of H_2_O_2_ at 240 nm); d = 1 cm (cuvette path length); and Vp = 0.04 mL (sample volume).

##### Glutathione Peroxidase Activity Assessment

Glutathione peroxidase (GPx) activity was determined using an indirect spectrophotometric method [39]. The method quantifies GPx activity by measuring the consumption of reduced glutathione (GSH) in the presence of peroxide. Unreacted GSH and peroxide reduce Cu(II)-neocuproine (Nc), forming a yellow-orange Cu(I)-Nc complex with maximal absorbance at 450 nm. Therefore, GPx activity is inversely proportional to the absorbance at 450 nm. The reaction mixtures, prepared in triplicate, contained 100 mM sodium phosphate buffer (pH 7.0), 4 mM GSH, the sample, and 2 mM H_2_O_2_ in a total volume of 2.0 mL. After a 10 min incubation at 37 °C, the reaction was terminated with trichloroacetic acid, and the supernatant was reacted with a freshly prepared CUPRAC reagent [Cu(II):Nc:NH_4_Ac; 1:1:1 (*v*/*v*/*v*)]. Following 30 min of incubation, the absorbance was measured at 450 nm using 96-well microplates and a Synergy 2 Multi-Mode Microplate Reader (BioTek Instruments, Inc., Winooski, VT, USA). GPx activity was expressed as units per liter (U/L), with one unit defined as the amount of enzyme catalyzing the oxidation of 1.0 μmol of GSH per minute at 25 °C and pH 7.0 [40].

#### 2.5.2. Determination of Inflammatory Biomarkers

The selection of the inflammatory cytokines IL-1β, IL-6, IL-8, and TNF-α as key points in this RCT is predicated on the established role of chronic, low-grade inflammation in the pathogenesis of MetS [7] in the selected at-risk population. Given that walnuts are a source of bioactive compounds, including omega-3 fatty acids and polyphenols, with putative anti-inflammatory properties, the measurement of these cytokines provides a direct assessment of the intervention’s capacity to modulate systemic inflammation, a critical factor in the context of MetS risk mitigation [29,41,42].

The anti-inflammatory capacity of walnut kernels was evaluated by measuring the concentrations of four inflammatory biomarkers, IL-1β, IL-6, IL-8, and TNF-α, in serum samples using commercially available 96-well microplates, with the Human IL-1β ELISA kit (Cat. No. EH0185), Human IL-6 ELISA kit (Cat. No. EH0201), Human IL-8 ELISA kit (Cat. No. EH0205), and Human TNF-α ELISA kit (Cat. No. EH0302) (FineTests Biotech Inc., Wuhan, China) were utilized in accordance with the manufacturer’s instructions using the Synergy 2 Multi-Mode Microplate Reader (BioTek Instruments, Inc., Winooski, VT, USA). For all biomarkers tested, the results were reported in picograms per milliliter (pg/mL).

### 2.6. Statistical Analysis

Data were analyzed using R software (version 4.3.2, R Foundation for Statistical Computing, Vienna, Austria). Descriptive statistics included means ± standard deviations (SD) for continuous variables and frequencies for categorical data. Linear mixed models assessed changes from baseline to final values, with intervention (walnut vs. control), period (first/last), and their interaction as independent variables, and a random effect for patients. Coefficients, 95% confidence intervals (CI), and *p*-values were reported; *p* < 0.05 (two-tailed) was considered significant for all analyses.

### 2.7. Ethical Considerations

Ethical approval was obtained from the Iuliu Hatieganu University of Medicine and Pharmacy Ethics Committee (AVZ 79/12 May 2023), and the study was conducted according to the Declaration of Helsinki, as previously reported [30]. All participants provided written informed consent, and data were anonymized. The trial was registered in ISRCTN (ISRCTN17119161) on 26 September 2024.

## 3. Results

Of 22 initial participants, 20 completed the study after one withdrawal (medical complications) and one exclusion (sample deterioration). The flow diagram can be found in the first published paper for this randomized controlled trial [30].

### 3.1. Baseline Characteristics

Table 1 summarizes the baseline characteristics of the 20 participants (47.67% female; mean age: 49.15 ± 4.11 years; range: 42–56).

Table 2 presents the baseline characteristics of all subjects at the start of each intervention period, providing a reference point for comparing subsequent changes in outcome measures.

### 3.2. Study Outcomes

First, we assessed the final characteristics of all subjects at the end of both intervention periods (Table 3).

Next, we computed the unadjusted changes from baseline for each outcome variable in the walnut and control groups (Table 4).

The primary effect measure was the change in each characteristic, calculated as the difference between the final and baseline values. To account for the complexity of the study design, linear mixed-effects models were employed. These models predicted changes in outcome variables as a function of treatment, adjusted for period, and included interactions and random effects for individual participants. The results are presented in Table 5.

The changes from baseline in TAC (*p* = 0.262), CAT (*p* = 0.676), and GPx (*p* = 0.943) indicated no statistically significant differences between the walnut and control groups. Similarly, the intervention did not have a significant impact on the pro-inflammatory cytokine levels: IL-1β (*p* = 0.092), IL-6 (*p* = 0.826), and TNF-α (*p* = 0.487). Although there was a trend towards increased IL-8 in the walnut group (LS Mean Difference: 4.8 pg/mL, 95% CI −0.94 to 10.55; *p* = 0.098), this effect did not achieve statistical significance.

Statistically significant changes were observed in the period adjustment (95% CI) for CAT [−15.15 (−29.38–−0.91); *p* = 0.038], GPx [137.82 (48.65–227); *p* = 0.003], and IL-1β [0.69 (0.05–1.33); *p* = 0.034], but not in the intervention/period interaction ratio (95% CI). For IL-8, after adjustments, a downward trend was observed in the intervention/period interaction ratio (95% CI) [−7.4 (−15.53–0.73); *p* = 0.073].

## 4. Discussion

### 4.1. Antioxidant Defense

#### 4.1.1. Total Antioxidant Capacity

In the context of TAC results, our findings are consistent with the results of a crossover RCT conducted by McKey et al. [43], which reported no significant within- or between-group changes in plasma antioxidant capacity, as measured by Oxygen Radical Absorbance Capacity (ORAC) and Ferric-Reducing Antioxidant Potential (FRAP). The study involved 21 generally healthy men and postmenopausal women aged ≥50 years, who consumed walnuts (21 g/day or 42 g/day) over a 6-week period, with a 6-week washout phase between intervention periods [43]. However, a four-period crossover RCT, which investigated the effects of acute consumption of whole walnuts (85 g), walnut pellicle (5.6 g), de-fatted walnuts (34 g), and walnut oil (51 g) on postprandial lipemia, endothelial function, oxidative stress, and cholesterol efflux in 15 overweight and obese adults (age range: 21–60 years) with moderate hypercholesterolemia, showed a significant treatment effect for FRAP, with higher FRAP values associated with walnut pellicle and oil treatments compared to de-fatted walnuts (*p* < 0.01) [44]. These divergent outcomes may be attributed to differences in study determination assays (the present RCT employed the TEAC assay, while the referenced studies utilized the FRAP assay), study design (acute consumption versus 4-week intervention duration), the quantity and form of administration of walnuts (both pellicle and oil have concentrated forms of polyphenols and PUFAs, tocopherols, and phytosterols, respectively, all of which have antioxidant properties [45,46,47,48]), and variations in study populations (knowing that the effects of bioactive compounds are stronger in unhealthy versus healthy individuals), which may influence responsiveness to walnut consumption.

#### 4.1.2. Catalase Activity

When comparing the findings of the current study with other crossover RCTs [49,50] notable differences emerge regarding CAT activity responses to walnut-enriched diets among populations at risk of CVD. In the present study, no significant difference in CAT activity was observed between the walnut-enriched group and the control group. However, Canales et al. reported a statistically significant increase in CAT activity, total glutathione, and oxidized glutathione (*p* < 0.05) in a placebo-controlled trial involving 22 volunteers (60% overweight and 40% obese) with elevated CVD risk [49]. Participants were randomly assigned to consume either walnut-enriched meat (20% walnut paste) or low-fat meat as a control over two 5-week intervention periods. Similarly, Sanchez-Muniz et al. observed a significant increase (*p* < 0.05) in CAT activity, along with SOD and paraoxonase-1 (PON-1) enzyme activity, following walnut interventions, particularly among individuals with specific PON-1 polymorphisms. Theis 5-week crossover RCT compared the antioxidant effects of low-fat meat versus walnut-enriched meat (20% walnut paste) in 22 overweight volunteers (mean age: 54.8 years) at high cardiovascular risk, stratified by different PON-1 192/55 polymorphisms [50]. The negligible changes in CAT activity observed in our study, on the one hand, may be due to the different population group (of the 20 participants in our study, 55% were overweight and 45% were obese) and, on the other hand, may reflect the complex interplay of genetic variability, intervention parameters, comorbidities, and baseline oxidative stress levels. These findings underscore the need for personalized dietary approaches and further investigation into genotype–diet interactions in modulating antioxidant responses.

Other dietary components such as prooxidant foods, may counteract the antioxidant effects of walnuts. Low levels of zinc or selenium could limit CAT responsiveness. Psychological stress or environmental pollutants can also elevate oxidative stress and potentially blunt the impact of dietary antioxidants.

#### 4.1.3. Glutathione Peroxidase Activity

The results of the present crossover RCT revealed no significant difference in GPx activity between the walnut and control groups. These outcomes align with those of a 19-week randomized crossover trial conducted by McKay et al. [43], which reported no significant changes in plasma antioxidant biomarkers, including GPx, following chronic walnut consumption (21 g/day or 42 g/day) in a cohort of 21 generally healthy men and postmenopausal women aged ≥50 years. The present results suggest that walnut consumption did not exert a statistically significant effect on GPx activity within this generally healthy population. However, the potential influence of confounding variables—such as baseline oxidative stress levels, individual health status, and methodological differences—highlights the need for further investigation. Future studies should aim to incorporate larger sample sizes, standardized dietary protocols, and stratified participant risk assessments to better elucidate the potential modulatory role of walnuts on oxidative stress markers, particularly in populations with elevated cardiovascular risk.

### 4.2. Biomarkers of Inflammation

#### 4.2.1. Interleukin-1 β

The data revealed a slight decrease in IL-1β levels in the control group and a minimal increase in the walnut group. Although the *p*-value of 0.092 suggests a trend toward a difference, it did not reach statistical significance. The results are in agreement with the outcomes of other studies which also reported no statistically significant effect on IL-1β levels after walnut consumption [51,52]. In the crossover RCT by Chiang et al., 25 participants (age range: 23–65 years) with normal to mildly hyperlipidemic profiles were assigned to three dietary interventions over 4 weeks: a walnut diet (42.5 g of walnuts, 6 times per week; 1.8% of energy from *n*-3 fat), a fish diet (2 times per week; 0.8% of energy from *n*-3 fat), and a control diet (no nuts or fish; 0.4% of energy from *n*-3 fat) [51]. Similarly, the crossover RCT by Burns-Whitmore et al. included a small sample size (*n* = 20) of healthy lacto-ovo-vegetarians, a population that may already consume a diet low in inflammatory markers, potentially masking the effects of walnut supplementation [52].

In contrast, Cofan et al. reported a significant reduction in IL-1β levels (standardized mean difference [SMD] = −0.1; 95% CI: −0.16 to −0.04, *p* < 0.001), suggesting that walnuts (30–60 g/day), rich in alpha-linolenic acid (ALA) and polyphenols, may exert anti-inflammatory effects [42]. The long duration of this parallel RCT study (2 years) and the large sample size (*n* = 634) showed the robustness of the findings, particularly in the context of healthy older adults (mean age: 69.1 ± 3.6 years), a population at increased risk for chronic inflammation. Similarly, a 6-week crossover RCT demonstrated that a diet high in ALA, a key component of walnuts as already mentioned, significantly reduced IL-1β production in hypercholesterolemic subjects (*p* < 0.05), concluding that ALA could modulate inflammatory pathways by decreasing the production of pro-inflammatory cytokines [53].

The variability in outcomes across studies underscores the influence of population-specific effects and intervention parameters. Cofan et al. [42] observed significant anti-inflammatory effects in older adults, a population prone to chronic low-grade inflammation, after using higher walnut doses (30–60 g/day) over a long time period (2 years). In contrast, Burns-Whitmore et al. [52] reported limited effects in healthy vegetarians with lower baseline inflammation after lower dose (28.4 g/day) treatments. Correspondingly, Zhao et al. [53] noticed significant changes in hypercholesterolemic individuals, suggesting that metabolic dysregulation enhances responsiveness. Dosage variations imply a dose–response relationship, particularly for IL-1β reduction, with bioactive components such as ALA potentially mediating these effects.

#### 4.2.2. Interleukin-6

The results of the present study revealed no significant difference in IL-6 levels between the walnut and control groups. These findings align with several RCTs that also reported no significant effect on IL-6 levels after walnut consumption. For instance, Aronis et al. found no significant change in IL-6 levels following a short-term (4-day) walnut-enriched diet (48 g/day) in 15 individuals (mean age: 58 ± 2.5 years) with metabolic syndrome (*p* > 0.05) [54]. Likewise, two other trials reported no significant effect of walnut consumption on IL-6 levels in healthy lacto-ovo-vegetarians and normal to mildly hyperlipidemic individuals, respectively (*p* = 0.23 and *p* > 0.05, respectively) [51,52].

However, in contrast to our outcomes, several other RCTs reported significant reductions in IL-6 levels with walnut consumption. For example, Cofan et al. conducted a large-scale, 2-year parallel RCT involving 634 healthy older adults and found a significant reduction in IL-6 levels with walnut consumption (standardized mean difference [SMD] = −0.18; 95% CI: −0.00 to −0.03; *p* < 0.001) [42], while Zhao et al. reported lower IL-6 production by peripheral blood mononuclear cells (PBMCs) in hypercholesterolemic subjects following a diet high in ALA (*p* < 0.05) [53]. Fatahi et al. also observed a significant reduction in IL-6 levels in overweight and obese women following a walnut-enriched weight-reducing diet (*p* < 0.001) [55]. Additionally, some other studies further supported the anti-inflammatory potential of walnuts, with significant reductions in IL-6 levels observed in overweight/obese individuals and high-risk CVD participants (*p* < 0.01 and *p* ≤ 0.04, respectively) [56,57]

These findings suggest that after walnut intake, IL-6 anti-inflammatory effects may not be universally noticed across all populations and study designs. Outcome variability may stem from study duration, population, dosage, and design. Longer studies (2–5 years, e.g., Cofan et al., 2020 [42]) showed pronounced anti-inflammatory effects, while shorter ones (4 days–8 weeks, e.g., Aronis et al., 2012 [54]) might be insufficient to significantly change the biomarker values. Populations with higher baseline inflammation (e.g., older adults, hypercholesterolemic individuals) exhibited stronger responses compared to relatively healthy people. Dosage variations (e.g., 30–60 g/day vs. 45 g/day) suggested a dose–response relationship, while parallel designs could provide more robust data than crossover studies.

#### 4.2.3. Interleukin-8

The present study observed a slight increasing trend in interleukin-8 (IL-8) levels in the walnut group, compared to a decrease in the control group. These results are consistent with previous research indicating that short-term walnut intake may not significantly alter inflammatory markers such as IL-8. For instance, Aronis et al. conducted a double-blind, randomized, placebo-controlled study, as mentioned above, and found that a short-term walnut-enriched diet (48 g/day) did not affect markers of inflammation, including IL-8 (*p* > 0.05), or vascular injury in obese individuals with MetS [54].

#### 4.2.4. Tumor Necrosis Factor-α

Our trial found no significant difference in TNF-α levels between the walnut and control groups. These findings align with several RCTs that also reported no significant effect of walnut consumption on TNF-α levels. Aronis et al. observed no significant changes in TNF-α levels following a short-term (4-day) walnut-enriched diet (48 g/day) in individuals with metabolic syndrome (*p* > 0.05) [54], while Chiang et al. did not notice a significant effect from a walnut diet on TNF-α levels in normal to mildly hyperlipidemic individuals over a 4-week period (*p* > 0.05) [51].

In contrast, the results of other RCTs reported significant reductions in TNF-α levels following walnut consumption. Cofan et al. conducted a parallel RCT and found a significant reduction in TNF-α levels after 2 years of daily walnut consumption (standardized mean difference [SMD] = −0.31; 95% CI: −0.54 to −0.08; *p* = 0.009) [42]. Zhao et al. detected lower serum TNF-α concentrations in hypercholesterolemic subjects following a diet high in ALA (*p* < 0.08) [53]. Similarly, Fatahi et al. reported a significant reduction in TNF-α levels in overweight and obese women following a walnut-enriched weight-reducing diet (*p* = 0.01) [55].

These results clearly suggest that the anti-inflammatory effects of walnuts on TNF-α may not be recognized in all populations and different study designs. The inconsistent findings could be explained by key methodological and contextual differences. Longer intervention periods (e.g., 2 years in Cofan et al. [42]; 12 weeks in Fatahi et al. [55]) may allow for more sustained anti-inflammatory effects compared to shorter periods of time (e.g., 4 days–4 weeks in Aronis et al. [54] and Chiang et al. [51]), suggesting that long-term daily walnut intake might be necessary for the onset of significant anti-inflammatory changes. Populations with elevated baseline inflammation, such as older adults or those with metabolic disorders, tend to show more significant responses, whereas healthier individuals exhibit minimal effects. As previously mentioned, variations in walnut dosage suggested a potential dose–response relationship. While study design—parallel versus crossover—can influence outcomes, each offers unique advantages. Parallel designs are typically preferred when carryover effects are a concern, whereas crossover designs can offer greater statistical efficiency when appropriate. These factors collectively account for the perceived variability in the results.

## 5. Strengths, Limitations, and Future Prospects

To the best of our knowledge, this is the first RCT to investigate the effects of short-term (4-week) daily walnut consumption on middle-aged adults at risk of MetS. This study benefited from a rigorous crossover design, enhancing statistical power by minimizing inter-individual variability, and a one-month washout period to reduce carryover effects. However, the strengths are balanced by several limitations. The unblinded design introduced potential for bias, and the four-week intervention period proved to be insufficient to elicit significant changes in the measured outcomes. The short duration could also explain the lack of significant findings; individual variability in metabolic response may mask subtle effects. However, the 4-week period was selected to optimize feasibility—supporting participant adherence, providing early information on dietary effects, and establishing a baseline for future long-term investigations. The relatively small sample size further limited statistical power, and the lack of a detailed phytochemical analysis of the walnut samples restricted the comparability of these findings to other studies. Furthermore, the administration of the intervention periods during different months (September vs. November) could have introduced a potential confounding factor, as seasonal variations in diet, physical activity, and sunlight exposure might independently influence the oxidative and inflammatory status of participants. The potential reduction in walnut absorption associated with age-related decline in gastric acid and enzyme activity could be considered a limitation; however, our participants were middle-aged adults with a mean age of 49.15 years (range 42–56) with less pronounced digestion issues compared to older populations. To optimize bioactive compound availability, future studies should consider using processed walnut products to address absorption variability in older individuals. While acknowledging these limitations, our RCT provides valuable data, adding to the growing, yet often conflicting, body of evidence regarding the impact of walnut consumption on cardiometabolic health in middle-aged adults. To further elucidate the immunomodulatory effects of walnuts, future research could explore alternative inflammatory or vascular markers and extend the duration of dietary interventions. Such approaches may provide a more comprehensive understanding of the potential role of walnuts in modulating inflammatory responses across diverse populations.

## 6. Conclusions

The present RCT, targeting a group of middle-aged adults at risk of developing MetS, shows that short-term (4-week) daily walnut consumption did not significantly alter oxidative stress and inflammation parameters, although it may contribute to maintaining cellular homeostasis. Further research is necessary to examine the effects of daily nut consumption over an extended duration (>3 months) on oxidative and inflammatory status in this population, as well as its potential for preventing or delaying the development of MetS. Additionally, exploring the mechanisms by which walnuts influence metabolic health could lead to helpful dietary recommendations for at-risk individuals. Understanding the long-term benefits of incorporating walnuts into the daily diet may enhance preventive strategies against MetS and improve overall health outcomes.

## Figures and Tables

**Table 1:** Baseline characteristics of participants by sequence.

Variables	Control ^a^-Walnut ^b^ (*n* = 9)	Walnut ^a^-Control ^b^ (*n* = 11)	*p*-Value
Age (years)	49.33 (4.21)	49.00 (4.22)	0.862
Female, *n* (%)	4.00 (44.44)	6.00 (54.55)	1.000
TAC (mmol/L)	0.39 (0.07)	0.46 (0.11)	0.127
CAT (U/mL)	10.51 (11.56)	9.80 (5.21)	0.857
GPx (U/L)	120.60 (68.42)	153.93 (58.72)	0.256
IL-1β (pg/mL)	1.48 (1.06)	1.14 (0.43)	0.345
IL-6 (pg/mL)	2.36 (1.24)	1.04 (0.55)	0.005
IL-8 (pg/mL)	24.75 (16.24) *	16.30 (8.42)	0.156
TNF-α (pg/mL)	1.70 (0.28)	1.67 (0.25)	0.812

Data are expressed as means (SD), except for qualitative variables, which are expressed as *n* (%). CAT—catalase; GPx—glutathione peroxidase; IL-1β—interleukin-1β; IL-6—interleukin-6; IL-8—interleukin-8; TAC—total antioxidant capacity; TNF-α—tumor necrosis factor alpha. * One sample was removed due to accidental damage (*n* = 8). ^a^—first intervention period; ^b^—second intervention period.

**Table 2:** Baseline characteristics for all subjects at the beginning of the two intervention periods.

Variables	Control (*n* = 20)	Walnut (*n* = 20)	*p*-Value
TAC (mmol/L)	0.41 (0.08)	0.43 (0.11)	0.445
CAT (U/mL)	15.88 (13.04)	12.73 (9.31)	0.384
GPx (U/L)	252.34 (132.35)	250.75 (126.84)	0.969
IL-1β (pg/mL)	1.18 (0.75)	1.10 (0.35)	0.693
IL-6 (pg/mL)	2.69 (5.51)	1.69 (1.39)	0.439
IL-8 (pg/mL) *	26.43 (26.45)	17.60 (7.41)	0.158
TNF-α (pg/mL)	1.69 (0.28)	1.72 (0.25)	0.735

Data are expressed as means (SD) for all quantitative variables. CAT—catalase; GPx—glutathione peroxidase; IL-1β—interleukin-1β; IL-6—interleukin-6; IL-8—interleukin-8; TAC—total antioxidant capacity; TNF-α—tumor necrosis factor alpha. * One sample was removed due to accidental damage (*n* = 19).

**Table 3:** Final characteristics for all subjects at the end of the two intervention periods.

Variables	Control (*n* = 20)	Walnut (*n* = 20)	*p*-Value
TAC (mmol/L)	0.40 (0.11)	0.39 (0.14)	0.875
CAT (U/mL)	14.88 (11.36)	13.82 (9.09)	0.746
GPx (U/L)	133.86 (205.57)	116.97 (193.52)	0.790
IL-1β (pg/mL)	1.01 (0.31)	1.12 (0.38)	0.331
IL-6 (pg/mL)	1.56 (1.68)	1.31 (1.63)	0.625
IL-8 (pg/mL) *	21.20 (8.62)	18.77 (6.19)	0.312
TNF-α (pg/mL)	1.72 (0.29)	1.80 (0.23)	0.378

Data are expressed as means (SD) for all quantitative variables. CAT—catalase; GPx—glutathione peroxidase; IL-1β—interleukin-1β; IL-6—interleukin-6; IL-8—interleukin-8; TAC—total antioxidant capacity; TNF-α—tumor necrosis factor alpha. * One sample was removed due to accidental damage (*n* = 19).

**Table 4:** Unadjusted change (final–baseline) in participants’ outcomes in walnut and control groups.

Variables	Control (*n* = 20)	Walnut (*n* = 20)	Difference Walnut–Control (95% CI)	*p*-Value
TAC (mmol/L)	−0.01 (0.13)	−0.04 (0.17)	−0.03 (−0.13; 0.06)	0.512
CAT (U/mL)	−1.00 (18.99)	1.09 (13.15)	2.09 (−8.37; 12.55)	0.688
GPx (U/L)	−118.48 (109.72)	−133.79 (127.22)	−15.30 (−91.35; 60.75)	0.686
IL-1β (pg/mL)	−0.16 (0.84)	0.02 (0.58)	0.18 (−0.29; 0.64)	0.441
IL-6 (pg/mL)	−1.13 (5.80)	−0.39 (1.65)	0.74 (−1.99; 3.47)	0.587
IL-8 (pg/mL) *	−5.23 (25.22)	1.17 (5.45)	6.40 (−5.28; 18.08)	0.274
TNF-α (pg/mL)	0.03 (0.42)	0.08 (0.31)	0.04 (−0.19; 0.28)	0.711

Data are expressed as mean (SD) change (difference between the final and baseline values) for all qualitative variables. The last column presents the difference between changes for the control and walnut groups. CAT—catalase; CI—confidence interval; GPx—glutathione peroxidase; IL-1β—interleukin-1β; IL-6—interleukin-6; IL-8—interleukin-8; SD—standard deviation; TAC—total antioxidant capacity; TNF-α—tumor necrosis factor alpha. * One sample was removed due to accidental damage (*n* = 19).

**Table 5:** Linear mixed models predicting change (difference between final and baseline values) in outcome variables as a function of treatment, adjusted for period, with interactions and random effects for participants (*n* = 20).

Dependent Variable	Intervention (95% CI)	*p*-Value	Period (95% CI)	*p*-Value	Intervention: Period (95% CI)	*p*-Value
TAC (mmol/L)	−0.08 (−0.21; 0.06)	0.262	−0.10 (−0.23; 0.04)	0.160	0.08 (−0.14; 0.30)	0.456
CAT (U/mL)	−2.95 (−17.18; 11.29)	0.676	−15.15 (−29.38; −0.91)	0.038	7.84 (−12.29; 27.97)	0.434
GPx (U/L)	−3.18 (−92.35; 85.99)	0.943	137.82 (48.65; 227.00)	0.003	3.69 (−131.48; 138.86)	0.956
IL-1β (pg/mL)	0.54 (−0.09; 1.18)	0.092	0.69 (0.05; 1.33)	0.034	−0.66 (−1.59; 0.27)	0.159
IL-6 (pg/mL)	−0.43 (−4.34; 3.49)	0.826	−2.40 (−6.32; 1.52)	0.221	2.05 (−3.68; 7.79)	0.471
IL-8 (pg/mL) *	4.80 (−0.94; 10.55)	0.098	4.68 (−1.06; 10.43)	0.107	−7.40 (−15.53; 0.73)	0.073
TNF-α (pg/mL)	0.12 (−0.22; 0.46)	0.487	0.15 (−0.19; 0.49)	0.370	−0.13 (−0.61; 0.35)	0.583

Data are expressed as means (SD) for all quantitative variables. CAT—catalase; CI—confidence interval; GPx—glutathione peroxidase; IL-1β—interleukin-1β; IL-6—interleukin-6; IL-8—interleukin-8; SD—standard deviation; TAC—total antioxidant capacity; TNF-α—tumor necrosis factor alpha. * One sample was removed due to accidental damage (*n* = 19).

## Data Availability

The original contributions presented in this study are included in the article. Further inquiries can be directed to the corresponding author.

## Abbreviations

The following abbreviations are used in this manuscript: ABTS—2,2-azinobis(3-ethylbenzothiazoline-6-sulfonate; ALA—alpha-linolenic acid; ARDs—age-related diseases; CAT—catalase; CI—confidence interval; CVD—cardiovascular disease; FRAP—Ferric-Reducing Antioxidant Potential; GPx—glutathione peroxidase; GSH—reduced glutathione; IL-1β—interleukin-1β; IL-6—interleukin-6; IL-8—interleukin-8; LS—least squares; MetS—metabolic syndrome; MyD88—myeloid differentiation primary response 88; Nc—Cu(II)-neocuproine; OS—oxidative stress; PON-1—paraoxonase-1; PUFAs—polyunsaturated fatty acids; RCT—randomized controlled trial; ROS—reactive oxygen species; SD—standard deviation; SOD—superoxide dismutase; T2D—type 2 diabetes; TAC—total antioxidant capacity; TLR4—toll-like receptor 4; TNF-α—tumor necrosis factor alpha; Trolox—6-hydroxy-2,5,7,8-tetramethylchroman-2-carboxylic acid; sVCAM-1—soluble vascular cellular adhesion molecule-1.
